# Heterologous prime–boost Zika virus vaccination induces comprehensive humoral and cellular immunity in mouse models

**DOI:** 10.3389/fimmu.2025.1578427

**Published:** 2025-04-25

**Authors:** Giuditta De Lorenzo, Rapeepat Tandavanitj, Lorena Preciado-Llanes, Ricardo Sanchez-Velazquez, Raissa Prado Rocha, Young Chan Kim, Arturo Reyes-Sandoval, Arvind H. Patel

**Affiliations:** ^1^ MRC-University of Glasgow Centre for Virus Research, Glasgow, United Kingdom; ^2^ Research and Technology Institute, Area Science Park, Trieste, Italy; ^3^ Biologicals Research Group, Research and Development Institute, Government Pharmaceutical Organization, Bangkok, Thailand; ^4^ The Jenner Institute, Nuffield Department of Medicine, University of Oxford, Oxford, United Kingdom; ^5^ Division of Structural Biology, Wellcome Centre for Human Genetics, University of Oxford, Oxford, United Kingdom; ^6^ Oxford Vaccine Group, Department of Paediatrics, Centre for Clinical Vaccinology and Tropical Medicine, Churchill Hospital, Oxford, United Kingdom; ^7^ Unidad Adolfo López Mateos, Instituto Politécnico Nacional (IPN), Mexico City, Mexico; ^8^ CVR-CRUSH, MRC-University of Glasgow Centre for Virus Research, Glasgow, United Kingdom

**Keywords:** ZIKV, vaccine, ChAdOx1, flavivirus, heterologous immunization

## Abstract

Zika virus (ZIKV) remained poorly studied until an outbreak in 2015 linked the virus to severe neurological disorders and congenital malformations. Currently, there are no antiviral drugs or vaccines available. We have previously demonstrated that a simian adenovirus vector vaccine (ChAdOx1 prMEΔTM) and a virus-like particle-based vaccine bearing E proteins locked in covalent dimers (VLP-cvD) are effective against ZIKV infection in animal challenge models. In this study, we further explored the efficacy of these vaccines, either individually or in combination, using a heterologous prime and boost vaccination strategy in mouse challenge models. Although the individual vaccines provided good protection levels, the heterologous prime–boost vaccination regimen (ChAdOx1 prMEΔTM followed by VLP-cvD) offered the most effective protection. This regimen elicited a strong cellular response and high levels of neutralising antibodies, which were attributed to ChAdOx1 prMEΔTM and VLP-cvD, respectively. Our findings support the use of combined vaccine technologies and offer valuable insights into the multifactorial protection achievable through heterologous vaccination. These results have important implications for the development of effective vaccination strategies against ZIKV and other emerging viruses.

## Introduction

Zika virus (ZIKV) is an enveloped RNA virus of the *Flaviviridae* family. Although initially associated with mild disease following its discovery in 1947 (1), recent outbreaks (between 2007 and 2015) in Yap Island, French Polynesia, and South America linked ZIKV infection to severe complications, including neurological disorders (Guillain–Barrè syndrome, encephalitis, myelitis) and congenital Zika syndrome (CZS) (2–6). The virus is primarily transmitted through the bite of infected mosquitoes, but sexual transmission (7) and vertical passage from mother to foetus (8) have also been reported. As a result, the World Health Organisation declared ZIKV a Public Health Emergency of International Concern, spurring increased efforts in vaccine development (9).

ZIKV has a single positive-strand RNA genome of 11 kb. Genetic differences distinguish its two major lineages, African and Asian (10). After entering infected cells, the genome is translated into a single polyprotein, which is cleaved by viral and cellular proteases into three structural proteins and seven nonstructural proteins (11, 12). The structural proteins include capsid (C), envelope (E), and membrane (M) protein, with the latter initially existing as a precursor membrane (prM) protein (11, 12). The immunodominant flavivirus E protein is the primary target of both neutralising and nonneutralising antibodies (13). The antigenic proximity between the E proteins of ZIKV and dengue virus (DENV) can lead to cross-reactive antibodies, which, if poorly neutralising, may contribute to antibody-dependent enhancement (ADE) of infection. As such, the risk of ADE presents a significant challenge in vaccine development (14–20). The E protein epitopes can be classified into three groups based on the neutralisation capacity of the binding antibodies: (i) ZIKV-specific epitopes, (ii) cross-reactive epitopes, and (iii) envelope dimer epitopes (EDEs) (13). ZIKV-specific antibodies mostly recognise the E domain III (DIII) or bind to complex quaternary epitopes on the protein. Broadly cross-reactive epitopes are mainly located on E domains I and II (DI and DII), particularly around the fusion loop peptide (FL). Although EDE-targeting antibodies are present in relatively small amounts, they exhibit strong neutralising capacity against both ZIKV and all four DENV serotypes. These antibodies bind the E protein in its dimeric conformation, thereby blocking the structural rearrangement required for membrane fusion and subsequent infection (21–24).

We utilise the clinically validated replication-deficient chimpanzee adenovirus vector (ChAdOx1) as a platform to engineer ZIKV vaccine candidates (25). A single dose of ChAdOx1 prMEΔTM, which encodes the ZIKV E protein with a deleted C-terminal transmembrane domain, cleared viraemia in a BALB/c mouse challenge model with Asian-lineage ZIKV-BR (25). In another study, using highly susceptible interferon receptor-deficient transgenic knockout A129 (Ifnar1^−/−^) mice, ChAdOx1 prMEΔTM reduced viral load in tissues and afforded 100% survival rate against challenge with the African-lineage ZIKV-AF (26).

More recently, we reported a viral-like particle (VLP) vaccine engineered to display the ZIKV E protein in a covalently linked dimeric conformation (cvD) to enhance E dimer exposure to the immune system (27). VLP-cvD conferred a 100% survival in A129 mice following lethal challenges with both ZIKV-BR and ZIKV-AF, significantly reducing viral dissemination to the brain and sexual organs (27). Furthermore, VLP-cvD immunisation blocked virus transmission from vaccinated mice to naïve mosquitoes feeding on them (28). Interestingly, in line with its superior protective efficacy, VLP-cvD elicited a higher proportion of antibodies sensitive to conformational changes than those in mice immunised with VLPs bearing the unmodified E protein (27), suggesting recognition of complex E epitopes. Both ChAdOx1 ZIKV prMEΔTM and VLP-cvD induced negligible DENV ADE in *in vitro* models (25, 27).

ChAdOx1 is a proven, effective, and safe vector vaccine platform in humans, as demonstrated by its global distribution as a coronavirus disease 2019 (COVID-19) vaccine. Even when administered as a single dose, nonadjuvanted ChAdOx-based vaccines have been shown to provide protection against disease and elicit strong humoral and cellular immune responses (29–36). However, to achieve long-lasting protection, many effective vaccines require multiple immunisations, typically through homologous boosting (37). This approach may be less suitable for viral vector-based vaccines, as antibodies generated against the vector could reduce the efficacy of repeated homologous doses. Individuals with high levels of preexisting antiadenovirus type 5 (Ad5) antibodies have been shown to produce lower antibody titres in response to the Ad5-based Ebola vaccine (38). To address this challenge, a heterologous prime–boost approach has been implemented for diseases such as malaria and tuberculosis, where expanding immune response breadth and improving vaccine effectiveness are critical (39, 40). In many cases, including the recent ChAdOx-based COVID-19 vaccination campaign, heterologous prime–boost regimens have demonstrated greater immunogenicity than homologous prime–boost strategies (41–45).

In this study, we evaluate the immunogenicity and efficacy of a heterologous vector prime and protein boost regimen using the ChAdOx1 prMEΔTM and VLP-cvD vaccine candidates in two mouse models to assess improvements in immunogenicity and protective efficacy against ZIKV infection.

## Results

### The humoral response elicited in BALB/c mice immunised with ChAdOx1 prMEΔTM and ZIKV VLP-cvD

**Figure 1:**
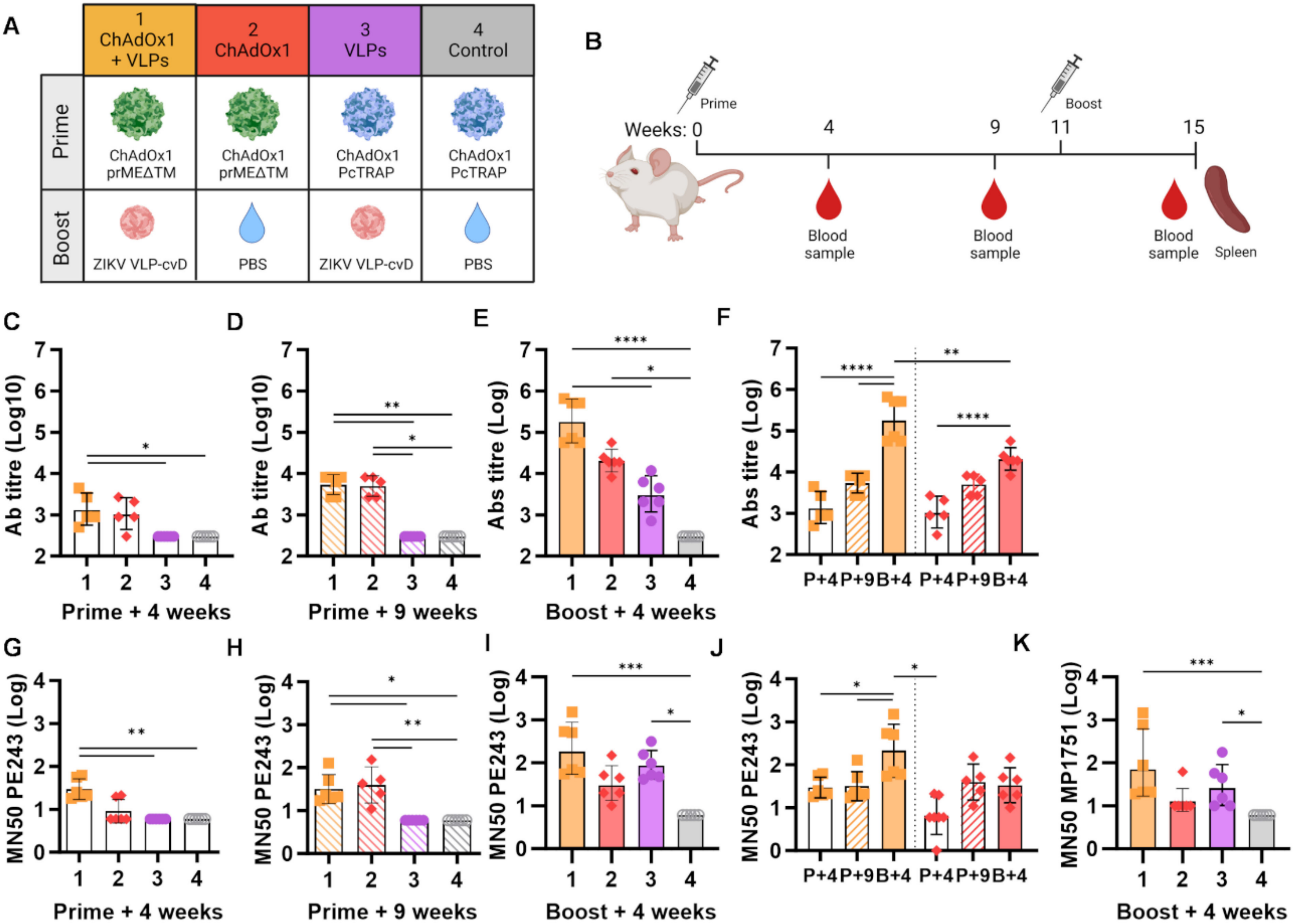
Humoral immunity induced by ChAdOx1 prMEΔTM and VLP-cvD in BALB/c mice. **(A)** Schematic representation of BALB/c mouse groups and ZIKV immunogen combinations used: ZIKV ChAdOx1 prMEΔTM prime and ZIKV VLP-cvD boost. ChAdOx1 encoding an unrelated malaria antigen (PcTRAP) and PBS were used as controls. **(B)** Immunisation protocol schematic. Each 6-week-old BALB/c mice (*n* = 6) received an intramuscular injection of ChAdOx1 prME ΔTM or the control ChAdOx1-malaria as a primary immunisation, followed by a subcutaneous boost with VLP-cvD or the control PBS at week 11. Blood samples were collected preboost at weeks 4 and 9. At week 15, animals were culled, and blood and spleens were collected. **(C–E)** Anti-E antibody titres in sera collected at the indicated time points from immunised animals, as shown in **(A)**. Antibody titres were determined using ELISA plates coated with biotinylated monomeric **(E)** The titre was defined as the maximum dilution that yielded a value higher than three times the value of the preimmune sera. Control sera were negative at the lowest dilution (1:900), and their titre was calculated as one-third of that dilution (300). **(F)** Data from animal groups 1 and 2 shown in **(C–E)** are plotted for comparison. Increase of anti-E titre in sera collected at weeks 4 (P+4, empty column), 9 (P+9, stripped column), and 15 (B+4, full column). **(G–I)** Neutralisation of PE243 ZIKV infection: serially diluted samples of mouse sera collected at the indicated time points were incubated with ZIKV PE243 for 1 h before infecting Vero-furin cells. At 72 h postinfection, the intracellular E levels were determined using capture sandwich ELISA, and the percentage infectivity relative to the virus alone was calculated. Results were plotted as MN_50_ values. **(J)** Data from animal groups 1 and 2 are shown in **(G–I)** plotted for comparison. Increase in neutralising titre in sera collected at week 4 (P+4, empty column), 9 (P+9, stripped column), and 15 (B+, full column) weeks. **(K)** Neutralisation of MP1751 ZIKV infection by postboost sera. Increase in ZIKV PE243 neutralisation by sera collected at the indicated time points. All graphs represent data combined from three independent experiments; columns show the geometric mean with geometric SD, and dots represent individual animals. Grouped lines correspond to the above-indicated asterisks.

Four groups of 6-week-old Balb/c (*n* = 6) were immunised as outlined in **Figure 1A**. Groups 1 and 2 received a primed intramuscular injection with 10^8^ IU of ChAdOx1 prMEΔTM, followed by a subcutaneous boost of 2 µg ZIKV VLP-cvD adjuvanted with AddaVax (group 1, ChAdOx1 + VLPs, orange) or PBS (group 2, ChAdOx1, red). Animals in groups 3 and 4 received 10^8^ IU of ChAdOx1 encoding an unrelated malaria antigen (PcTRAP) and were boosted with either adjuvanted ZIKV VLP-cvD (group 3, VLPs, purple) or PBS (group 4, negative control, grey). Booster immunisations were administered 11 weeks after the primary dose. Blood samples were collected at 4 and 9 weeks after primary immunisation, and mice were culled at 4 weeks postboost (i.e., 15 weeks after primary immunisation) for blood and spleen collection (**Figure 1B**).

Anti-ZIKV E antibody levels in the animal sera were measured by ELISA (**Figures 1C–F**). A modest antibody response was detectable in groups 1 and 2, which were primed with ChAdOx1 prMEΔTM, as early as 4 weeks, with levels increasing by week 9 (**Figures 1C, D**, orange and red). Four weeks after the boost, animals in the VLP-cvD-boosted group 1 showed a significant increase in anti-E antibody titres compared to the PBS-administered group 2 (**Figures 1E, F**, orange vs. red). Notably, a single dose of VLP-cvD (group 3) induced antibody levels comparable to those observed 4 weeks postprimary immunisation with ChAdOx1 prMEΔTM but significantly lower than the titres measured in the group that received both ChAdOx1 prMEΔTM and VLP-cvD (**Figure 1E**).

Sera samples were also used to quantify the neutralising activity of the elicited antibodies against the ZIKV strain PE243, a Brazilian isolate belonging to the Asian lineage (46), in cultured cells (**Figures 1G–J**). At 4 weeks postprime, neutralisation titres were modest in groups receiving ChAdOx1 prMEΔTM (**Figure 1G**), with no significant increase over time (**Figures 1H, J**). However, boosting with VLP-cvD (group 1) substantially increased neutralising antibody levels compared to those measured in the PBS-administered group (group 2) (**Figures 1I, J**). Animals in group 3, immunised with VLP-cvD but not ChAdOx1, exhibited neutralising titres comparable to those in the prime–boost group (**Figure 1I**). Similar results were obtained when measuring neutralising titres against the ZIKV strain MP1751, an African lineage with higher replication and virulence (47) (**Figure 1K**).

These data show that ChAdOx1 prMEΔTM elicits anti-ZIKV E antibodies that increase over time but without resulting in a proportional rise in neutralising antibody levels. In contrast, boosting with VLP-cvD results in an incremental increase in both antibody and neutralisation levels (**Figures 1F, J**). Interestingly, when administered alone, VLP-cvD elicits stronger humoral and neutralising antibody responses compared to ChAdOx1 prME ΔTM (group 3 vs. group 2, **Figure 1I**).

### Cellular responses elicited in BALB/c mice immunised with ChAdOx1 prMEΔTM and ZIKV VLP-cvD

**Figure 2:**
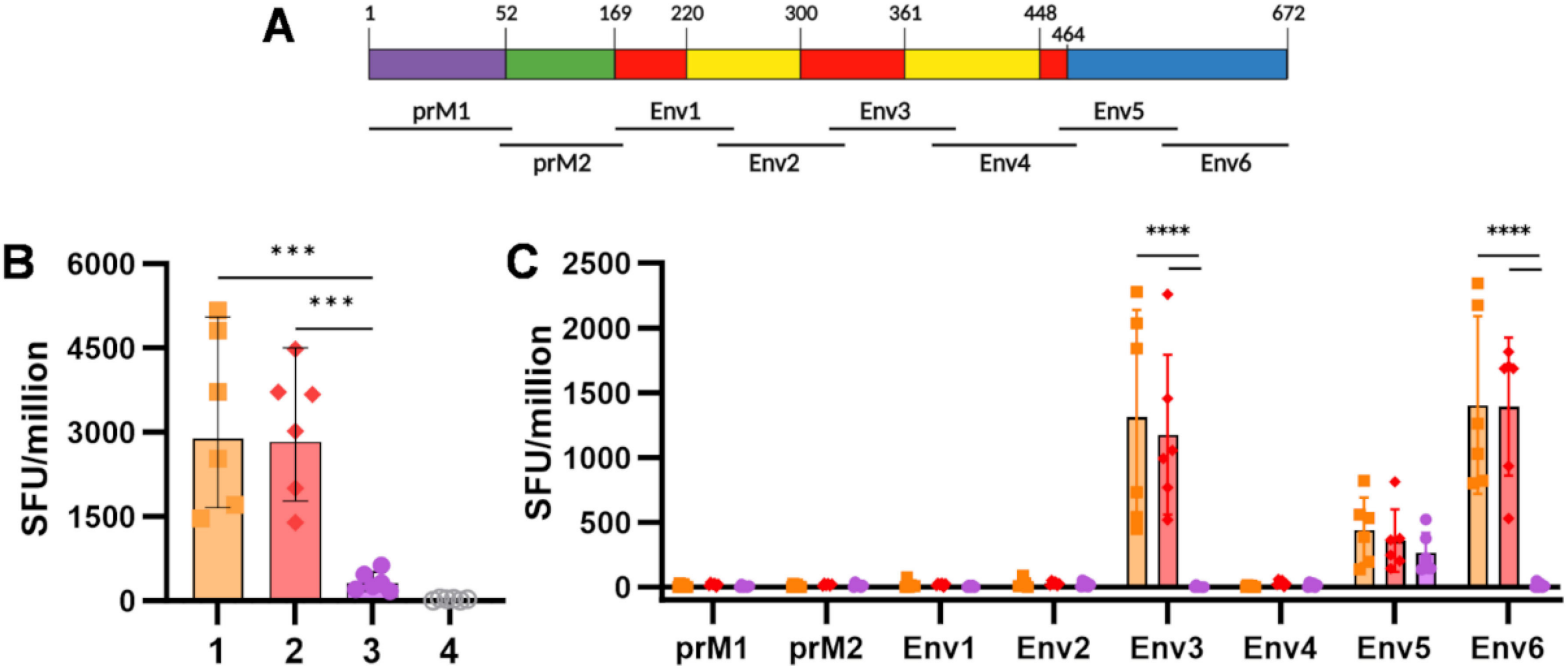
Cellular immunogenicity induced by ChAdOx1 prMEΔTM and VLP-cvD in BALB/c mice. **(A)** Schematic representation of the ZIKV prME polyprotein sequence, showing the location (relative to the polyprotein) of the six pools of overlapping 20-mer peptides utilised as stimulants in the ELIspot assay. **(B)** Cumulative spot-forming units (SFU) recorded per million splenocytes. **(C)** Number of SFU per individual peptide pool. Columns represent means, bars indicate SD, and dots represent individual animals. Grouped lines correspond to the above-indicated asterisks.

Animals were culled, and spleens were harvested 15 weeks after the prime immunisation. Isolated splenocytes were stimulated with eight pools of 20-mer overlapping peptides, spanning the entire prME sequence of ZIKV. Interferon-gamma (IFN-γ)-producing cells were quantified by ELISpot, and results were reported as spot-forming units (SFU) (**Figure 2A**). A similar cumulative SFU was measured in the two groups primed with ChAdOx1 prMEΔTM, showing no significant differences between the animals receiving VLP-cvD or PBS as a boost (**Figure 2B**, orange and red). Significantly fewer IFN-γ-producing cells were present in the group immunised with VLP-cvD alone (**Figure 2B**, purple). This observation, along with the lack of an increase in the number of SFU after the VLP boost, suggests that the VLP platform is a poor stimulator of cellular response.

Analysis of the cellular response against each separate peptide pool (**Figure 2C**) showed that ChAdOx1 prMEΔTM targets a specific IFN-γ response against aa 135–244 of E, spanning through DI and DII (Env3 pool) and against aa 405–504, corresponding to the C-terminal of DIII (Env6 pool). Some responses were also detected against aa 315–414, corresponding to the N-terminal of DIII (Env5 pool), but they were threefold lower. In contrast, animals immunised with VLP-cvD alone showed a modest but specific response to the N-terminal of the DIII region (Env5 pool) (**Figure 2C**, purple). The magnitude of this VLP-mediated cellular response was not sufficient to boost the SFU counts achieved by the ChAdOx1 prime (**Figure 2C**, yellow vs. red).

These data support the strong cellular immunogenicity induced by vector-mediated immunisation, with no additive effect observed after a protein-based boost.

### Immunogenicity assessment of ChAdOx1 Zika prME ΔTM and ZIKV VLP-cvD in A129 mice

**Figure 3:**
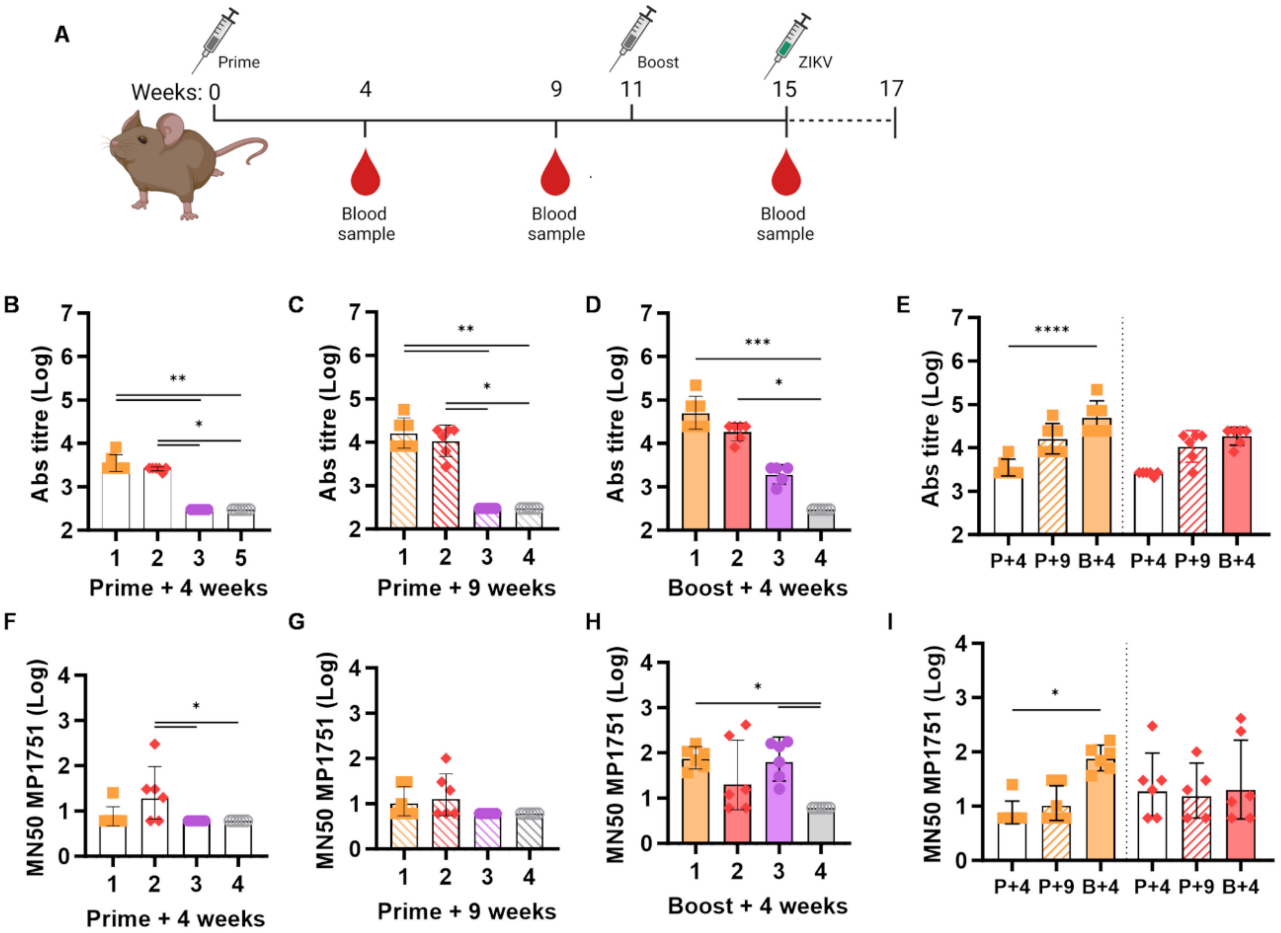
Humoral response in A129 mice. **(A)** Schematic of the immunisation protocol. Each group of the 4-week-old A129 (*n* = 6) mice was immunised as described in the **Figure 1** legend. At week 15 postimmunisation, following the collection of a prechallenge blood sample, animals received a subcutaneous injection with 100 PFU ZIKV MP1751 and were monitored for 14 days. **(B–D)** Anti-E antibody titres in sera collected at the indicated time points from immunised animals, as indicated in **(A)**. **(E)** Data of animal groups 1 and 2 in **(B–D)** plotted for comparison, showing the increase in neutralising titre in sera collected at weeks 4 (P+4, empty column), 9 (P+9, stripped column), and 15 (B+, full column). **(F–H)** Neutralisation of ZIKV MP1751 infection by mouse sera, as described previously. **(I)** Data from animal groups 1 and 2 in **(F–H)** plotted for comparison. Increase in neutralising titre in sera collected at weeks 4 (P+4, empty column), 9 (P+9, stripped column), and 15 (B+, full column). All graphs are based on data combined from three independent experiments, columns represent the geometric mean with geometric SD, and dots represent individual animals. Grouped lines correspond to the above-indicated asterisks.

To test the *in vivo* efficacy of our vaccines, we utilised Ifnar1^−/−^ A129 mice, which, unlike immune-competent BALB/c mice, are susceptible to ZIKV infection. Our immunisation schedule was similar to that used for BALB/c, with a vector prime followed by a protein boost at week 11, and the collection of two preboost and one postboost blood samples (**Figure 3A**). As shown in **Figures 3B, C**, sera collected from ChAdOx1 prMEΔTM-primed A129 mice during the preboost phase contained anti-ZIKV E antibody levels that were similar to those observed in BALB/c. Administration of a VLP-cvD boost also led to an increase in anti-E antibody levels in A129 mice (**Figures 3D, E**), although this increase was not as pronounced as that observed in BALB/c mice. We then tested the sera for their ability to neutralise the highly virulent ZIKV African lineage strain MP1751 in cultured cells using our microneutralisation assay. As shown in **Figures 3F–I**, we measured significant MN_50_ titres in the sera from animals in group 1, which were boosted with VLP-cvD following the ChAdOx1 prMEΔTM prime, but not in animals from group 2, which received PBS as a boost. We also observed significant levels of MP1751-neutralising antibodies in sera from animals in group 3, which were primed with the ChAdOx1 PcTRAP control vector vaccine and subsequently boosted with VLP-cvD (**Figure 3H**).

These data replicate our observations in BALB/c mice and confirm the capabilities of ChAdOx1 prMEΔTM to elicit high levels of anti-E antibodies and of VLP-cvD to generate a high proportion of neutralising antibodies.

### Assessment of vaccine efficacy in A129 mice challenged with ZIKV MP1751

**Figure 4:**
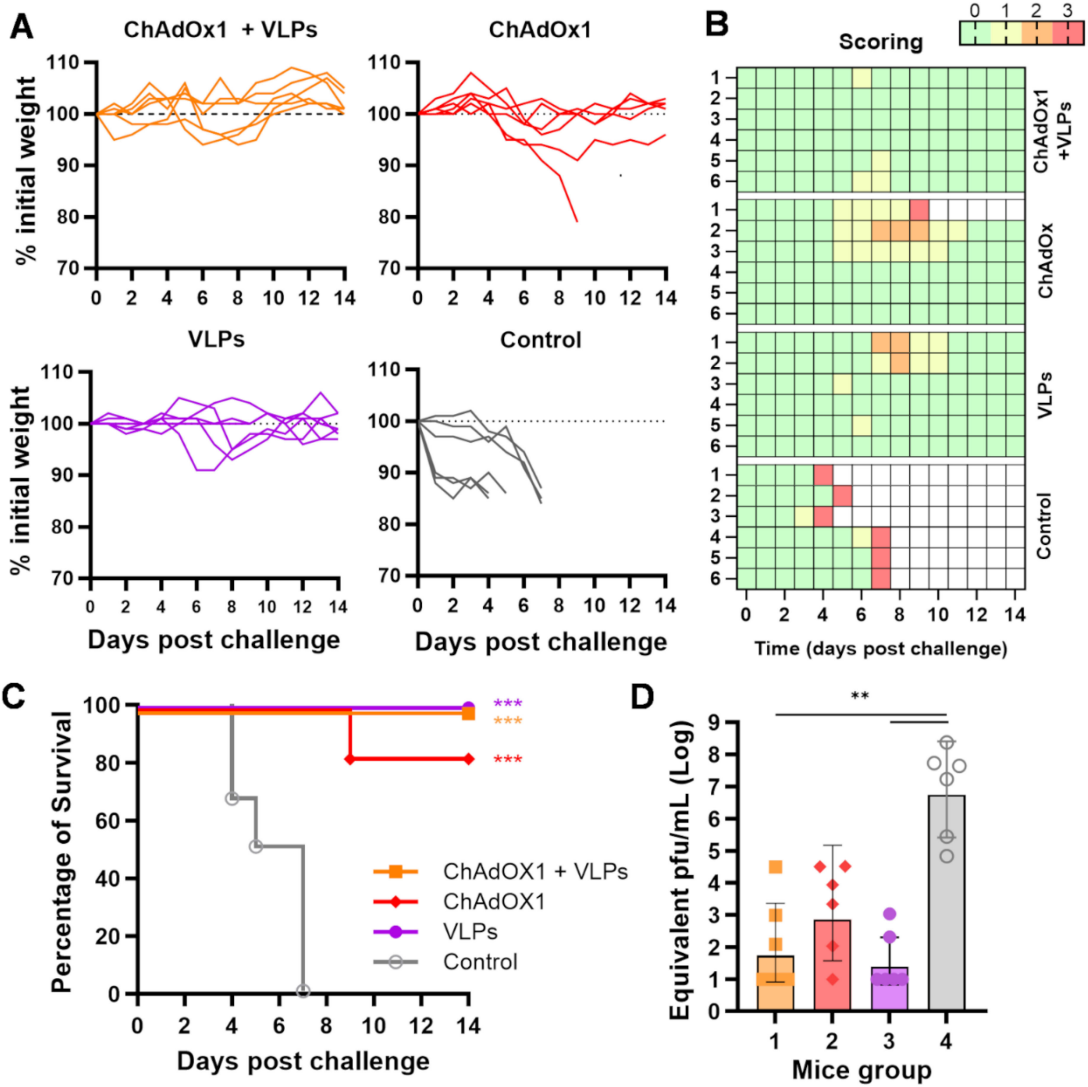
ZIKV MP1751 challenge. **(A)** All immunised animals described in **Figure 3** were challenged with live ZIKV MP1751 as outlined, and their body weights relative to preinfection levels were plotted. **(B)** Daily clinical scoring postchallenge. The scoring system used to monitor animal health following the ZIKV challenge is as follows: 0 (green) for no signs of distress or disease, 1 (yellow) for one sign of distress, 2 (orange) for two signs of distress or mild disease, and 3 (red, humane endpoint) for more than two signs of severe disease or 15% body weight loss. **(C)** Percentage of animal survival after challenge. **(D)** Viral titre in challenged animals. ZIKV levels in serum at 4 days postinfection were quantified by RT-qPCR and plotted as equivalent PFU per millilitre. The limit of quantification, estimated at 100 PFU/ml, is indicated by the dotted line. Columns represent the geometric mean with geometric SD, and dots represent individual animals. Grouped lines refer to the above-indicated asterisks.

To assess the efficacy of our immunisation regimes *in vivo*, we challenged the immunised A129 mice at week 15 (i.e., 1 month after the booster administration) with 100 plaque-forming units (PFU) of ZIKV MP1751. Animals were monitored for 14 days following infection for variations in body weight (**Figure 4A**) and the manifestation of clinical signs of infection, which were scored using a system ranging from 0 to 3 (**Figure 4B**). As expected, animals in the control group (group 4) quickly reached the humane endpoint (score 3) and were culled between days 4 and 7 postchallenge. Instead, all immunised animals (groups 1 to 3) survived the challenge, except for one individual in group 2 (**Figure 4C**). Some individuals in groups 2 and 3 showed signs of mild disease, receiving a score of 2 (**Figure 4B**). Group 1 animals showed generally good health, with sporadic signs of distress, receiving a score of 1 around days 6–7 postchallenge.

A blood sample was collected on day 4 postchallenge, a time point previously shown to correspond to the peak of viremia after ZIKV MP1751 infection (27, 28). The presence of ZIKV was quantified in sera by RT-qPCR. While the control animals showed high viremia, ranging from 10^5^ to 10^8^ equivalent PFU/ml, immunised mice exhibited an overall reduction in viral titre, though to varying degrees, with only the VLP-boosted groups showing a significant reduction (**Figure 4D**).

Results from this A129 challenge model indicate that all three immunisation regimes conferred good protective effects, with a better overall outcome in animals that received the heterologous prime–boost regimen.

## Discussion

The heterologous prime–boost approach consists of sequential immunisation programmes in which unmatched delivery methods or antigens are used. This combination often results in greater protective efficacy than multiple vaccinations with homologous antigens and may offer a substantial benefit for future vaccine design (48). In this study, we explored a heterologous vector prime and protein boost immunisation regimen against ZIKV using the ChAdOx1 prMEΔTM and VLP-cvD candidate vaccines, with the aim of improving their immunogenicity and efficacy. Our data showed that mice receiving ChAdOx1 prMEΔTM developed high anti-ZIKV E antibody levels, but both antibody and neutralisation titres increased significantly after boosting with VLP-cvD. On the contrary, ChAdOx1 prMEΔTM induced high T-cell responses, which were not elevated by VLP-cvD, indicating that the latter are poor stimulators of cellular immunity.

The concept of stimulating different arms of the immune system based on vaccine delivery methods emerged during the evaluation of HIV-1 vaccine candidates. Recombinant envelope protein elicited more protective antibodies but poor cytotoxic T-cell response, which were instead stimulated by vaccinia vector-mediated immunogen expression (49). A well-established concept suggests that neutralising antibodies offer a better chance of protecting against infection (50), while cellular-based immune responses are expected to play a stronger role in the long-term reduction of mortality and morbidity (51, 52). Our challenge data showed that, when expressed alone, ChAdOx1 prMEΔTM and VLP-cvD achieved good protective efficacy by stimulating different arms of the immune response. Nevertheless, a more comprehensive outcome can be achieved with a heterologous prime–boost regimen (ChAdOx1 prMEΔTM +VLP-cvD), combining the strong T-cell response induced by ChAdOx1 prMEΔTM and high virus neutralisation by VLP-cvD.

In our hands, VLP-cvD appeared to be a poor stimulator of cellular immunity, which is not uncommon for protein-based vaccines. Vaccine platforms such as vector-based or genetic vaccines are known to be stronger inducers of T-cell responses (53, 54). While ChAdOx1 prMEΔTM produced a strong cellular response toward two separate ZIKV E regions (amino acid (aa) residues 135–244 and 405–504), VLP-cvD induced a poor response to an area representing mostly E domain DIII (aa 315–414).

Considering the proximity between DENV and ZIKV, preexposure to one virus may influence, in terms of timing and magnitude, the response generated following immunisation with the other (55). Since the two flaviviruses share insect vectors and overlap in many geographical areas, this aspect is highly relevant for the development of Zika vaccines intended for populations exposed to DENV. Population studies have shown that ZIKV-infected individuals with no preexposure to DENV predominantly generate cellular responses targeting viral structural proteins, whereas nonstructural proteins are favoured in DENV-infected individuals (55). Interestingly, when ZIKV infection occurs in individuals already exposed to DENV, the T-cell response is less directed toward structural proteins, compared to the response in the DENV-negative individuals, suggesting a certain degree of immunodominance in the epitope profile. An investigation on preexisting ZIKV immunity in the DENV-exposed population highlighted 93 T-cell epitopes with potential for cross-reactivity between the two viruses, correlating cross-reactivity with sequence homology (56). Unfortunately, the relationship between T-cell immunodominance and cross-reaction in terms of disease severity remains unclear (56). Our ELISpot assay, conducted using splenocytes from DENV-naïve mice receiving candidate Zika vaccines, revealed a distinct T-cell epitope profile compared to that observed in DENV-exposed individuals, suggesting that the cellular response stimulated by our vaccine is ZIKV-specific. It raises an important question: Which T-cell response profile does the immunisation elicit in previously DENV-exposed individuals?

While cellular responses to DENV and ZIKV infection remain an underexplored area of research, there is a more comprehensive understanding of the nature of humoral responses to these viruses. In particular, for DENV, there is extensive knowledge of the role, both at structure–function levels, of the elicited antibodies (57). In our study, VLP-cvD boost increases the levels of anti-ZIKV E antibodies, including neutralising antibodies. However, it is noteworthy that VLP-cvDs alone reached the same neutralisation levels, despite showing much lower anti-ZIKV E antibody titres. The capacity of VLP-cvDs to elicit a higher percentage of neutralising antibodies is attributed to the specifically designed VLP-based immunogen, which displays ZIKV E in the covalent dimeric state in the prefusion conformation. This enhances the exposure of a potent class of virus-neutralising epitopes (EDE epitopes) (58). Unlike other flaviviruses, ZIKV does not always present this protein structure but rather a mixed population of mature and immature E proteins. This phenomenon reduces the likelihood of stimulating the production of EDE-binding antibodies, favouring the production of more abundant but less neutralising anti-DDII antibodies. The higher efficacy of VLP-cvDs was achieved by engineering the E protein with an A264C substitution, thus locking it in a covalently stabilised dimer. We previously demonstrated that VLP-cvDs specifically elicit antibodies that bind to complex, conformational-dependent epitopes, thereby conferring potent neutralising and protective efficacy (27).

The difference in immunogen design influences the profile of anti-E immunoglobulins elicited after immunisation, with ChAdOx1 prMEΔTM stimulating high antibody titres that do not correlate with high neutralisation. However, the production of high levels of nonneutralising antibodies should not be overlooked, as it represents a possible risk of stimulating ADE upon vaccination due to the similarity between ZIKV and DENV E proteins. The lack of reliable *in vitro/in vivo* models for ADE complicates the situation, preventing a comprehensive preclinical evaluation of these aspects. Therefore, it would be ideal to engineer the ChAdOx vaccine to directly produce VLP-cvD. Unfortunately, previous studies have shown that dimerisation is more efficiently achieved when the E protein is expressed at 28°C rather than at the physiological temperature of 37°C (59, 60). While temperature is a parameter that can be easily controlled during the *in vitro* production of protein-based vaccines, it cannot be regulated during vector or RNA-mediated protein expression. Therefore, including the A264C substitution in the ChAdOx1-based vaccine was deemed inappropriate, if not detrimental, prompting us to test the heterologous regimen involving unmatched antigens. Our VLP-cvD data are highly encouraging, demonstrating the benefit of rational engineering of the immunogen. Given that vector and RNA-based platforms are now well-established techniques, further efforts should focus on optimising the immunogen to overcome the temperature limitation for vector/RNA-based vaccines, fully harnessing their potential.

In conclusion, our analysis emphasises the importance of a deeper understanding of epitope characterisation, including both humoral and cellular determinants, to establish the foundation for optimal vaccine design and immunisation strategies capable of overcoming the specific challenges posed by the immunological interplay between ZIKV and DENV. Our study confirms the value of ChAdOx1 prMEΔTM and ZIKV VLP-cvD as vaccine candidates and, more importantly, highlights their potential to achieve multifactorial immunogenicity when applied in a heterologous prime–boost regimen. Through this approach, we demonstrate that the individual advantages of both vaccine platforms (low production cost and potential for engineering) can be combined to generate a comprehensive humoral and cell-mediated response, leading to more efficacious and long-lasting protection.

## Methods

### Animal ethics

All animal research described in this study was approved by the University of Glasgow Animal Welfare and Ethical Review Board and conducted under United Kingdom Home Office Licenses (P9722FD8E), in accordance with approved guidelines and the UK Home Office Animals (Scientific Procedures) Act 1986 (ASPA).

### Cell lines and virus strain

Expi293F embryonic human kidney cells were maintained and transfected in Expi293™ Expression Medium (Thermo Fisher Scientific, UK) according to the manufacturer’s protocol. Vero-furin cells, kindly supplied by Dr Theodore C. Pierson (61), were cultured in Dulbecco’s modified Eagle’s medium (DMEM) (Life Technologies, UK) containing 7% foetal bovine serum (FBS) (Life Technologies, UK), 10 μg/ml of blasticidin (InvivoGen, France), and penicillin–streptomycin (Gibco, UK). ZIKV PE243 (46) and ZIKV MP1751 (005V-02871; kindly supplied by Public Health England; accession number KY288905.1) were used for the microneutralisation assay. ZIKV MP1751 was used in animal challenge experiments.

### Design and production of the ChAdOx1 Zika vaccine

ChAdOx1 prME ΔTM was designed and produced as previously reported (25). The immunogenicity and efficacy profile of this vaccine in mice has been recently demonstrated (25, 26).

### VLP-cvD production and purification

VLP-cvD were produced in Expi293F cells at 28°C following transfection with a plasmid expressing ZIKV prM-E-A264C and purified by a combination of density gradient centrifugation and size-exclusion chromatography, as previously described (27). Eluted fractions were concentrated by ultrafiltration through Amicon^®^ Ultra 15 (100 kDa, Merck Millipore, USA). The concentration of the purified proteins was determined using a NanoDrop One (Thermo Fisher Scientific, UK).

### Mouse immunisation and ZIKV challenge

Six-week-old BALB/c mice (Envigo, UK) or 4-week-old A129 mice (129 Sv/Ev background; Marshall BioResources, UK) were immunised intramuscularly in each leg with 25 µl containing 10^8^ IU of ChAdOx1 Zika prME ΔTM or ChAdOx1 encoding an unrelated malaria antigen (PcTRAP). At 11 weeks from primary immunisation, mice were injected subcutaneously with either VLP-cvD adjuvanted with 2% AddaVax (InvivoGen, France) or adjuvants in PBS as a control. Prechallenge blood samples were collected for antibody titration and microneutralisation assays. The viral challenge was performed on A129 mice by subcutaneous injection of 100 PFU of MP1751 ZIKV in 100 µl of 2% FBS-DMEM. Postchallenge blood samples were collected for serum viral load. After the challenge, the mice’s weight changes and symptoms were monitored daily and scored for 14 days. Clinical scores were assigned according to our licence as follows: 0 for no signs of distress or disease, 1 for one sign of distress, 2 for two signs of distress or mild disease, and 3 for more than two signs of severe disease or loss of 15% of the body weight. A score of 3 was considered the humane endpoint, and mice were culled. Any individual mouse reaching a clinical score of 3 or losing more than 15% of the initial body weight was euthanised. Surviving mice were euthanised at 14 days postchallenge (dpc).

### ELISA for antibody titration

Recombinant *in situ* biotinylated ZIKV sE protein was expressed at 28°C using an ExpiFectamine 293 transfection kit (Thermo Fisher Scientific, UK), as described previously (27). Cell supernatant was harvested and dialysed. Biotinylated proteins were captured in ELISA plates precoated with 5 μg/ml of avidin (Sigma, UK) in Na_2_CO_3_-NaHCO_3_ buffer (pH 9.6) and subsequently blocked with PBS containing 0.05% Tween 20 (PBST) and 1% bovine serum albumin (BSA; Sigma, UK). Serial dilutions of mouse sera were tested for binding to the biotinylated proteins, and the bound antibodies were detected using horseradish peroxidase (HRP)-conjugated antimouse IgG A4416 (Sigma, UK) and 3,3′,5,5′-tetramethylbenzidine (TMB) substrate (Life Technologies, UK). Background signal was considered the average absorbance measured for the control/naïve sera. Antibody endpoint titres were calculated as the highest reciprocal serum dilution that resulted in an absorbance greater than threefold that of the background values.

### Microneutralisation assay

This assay was performed as previously described (25). Briefly, Vero-furin cells were seeded the day before the experiment at a density of 7 × 10^3^/well in 96-well plates. Threefold serially diluted mice sera were first incubated at 37°C for 1 h with 100 PFU/well of ZIKV. The serum/virus mix was then used to infect the cells. After 1 h of incubation at 37°C, 100 μl of medium was added to each well. At day 3 postinfection, cells were lysed in lysis buffer (20 mM Tris-HCl [pH 7.4], 20 mM iodoacetamide, 150 mM NaCl, 1 mM EDTA, 0.5% Triton X-100, and cOmplete protease inhibitors), and the viral E protein was quantitated by sandwich ELISA (see below). The amount of E protein detected correlates with the level of virus infectivity, which is presented as a percentage of ZIKV infectivity relative to the control (i.e., virus not preincubated with immune sera) (25). The MN_50_ titre was defined as the serum dilution that neutralised > 50% of ZIKV and was determined using GraphPad Prism 9. Nonlinear regression (curve fit) was performed for the data points using log (inhibitor) versus response (variable slope).

### Sandwich ELISA for quantification of ZIKV infectivity

ELISA plates were coated with 3 μg/ml of purified pan-flavivirus monoclonal antibody (MAb) D1-4G2-4-15 (ATCC HB112™, USA) in PBS, incubated overnight at room temperature (RT), and blocked for 2 h at RT with PBST and 2% skimmed milk powder. After washing with PBST, ZIKV-infected cell lysates were added and incubated for 1 h at RT. Wells were washed with PBST, incubated with anti-ZIKV E rabbit polyclonal R34 IgG^25^ at 6 μg/ml in PBST for 1 h at RT, and then washed again. Antibodies bound to the ZIKV envelope protein were detected using HRP-conjugated antirabbit IgG 7090 (Abcam, UK) and TMB substrate (Life Technologies, UK).

### ELISpot assay

ELISpot was carried out using splenocytes, following methods previously described (34), with some modifications. Briefly, ELISpot plates (Millipore MAIPS4510) were activated with 30% ethanol and coated overnight with antimouse IFN-γ mAb clone AN18 (3321-3-1000 Mabtech, UK, 5 μg/ml). Plates were washed and blocked for 2 h with RPMI media containing 10% FBS. Splenocytes (2.5 × 10^5^ per well) were stimulated with ZIKV-specific 20-mer peptides, overlapping by 10 amino acids (at 10 μg/ml), spanning the premembrane and envelope regions of ZIKV. After 16 h incubation, cells were discarded, and plates were washed with PBS. Antimouse IFN-γ mAb R46A2 biotinylated (3321-6-1000 Mabtech, UK, 1:1,000 dilution) was added to each well and incubated for 2 h. After washing, plates were incubated with streptavidin alkaline phosphatase reagent (3310-10-1000 Mabtech, UK, 1:1,000 dilution) for 1 h. After another washing step, development buffer BCIP/NBT(Plus) (Europa Bioproducts, UK No. MO711A) was applied. Once spots became visible, the reaction was stopped by washing the plate with water. Spots were acquired using an ELISPOT reader (Iris, Mabtech, UK). SFU/10^6^ splenocytes producing IFN-γ were calculated.

### RT-qPCR for mouse viremia

Viral RNA was extracted from postchallenge sera using the QIAamp^®^ viral RNA mini kit (QIAGEN, UK). Viral load was measured by RT-qPCR using the One-Step SYBR^®^ Primescript™ RT-PCR kit II (Takara, UK). CT values from serum samples were used to calculate the serum viral load according to a regression equation built from a set of standard viral RNA extracted from dilutions of a known titre virus preparation. The primers for the *MP1751 ZIKV* gene were as follows: Forward:5′-ACTTCCGGTGCGTTACATGA-3′ and Reverse:5′-GGGCTTCATCCATGATGTAG-3′.

### Statistical analysis

All statistical analyses were produced using GraphPad Prism 9 (GraphPad Software Inc, San Diego, CA, USA). A Shapiro–Wilk test was used to assess the normality of data distributions, and parametric and nonparametric tests were selected accordingly for antibody, neutralisation, and viral titres, based on log-transform data. Statistical analysis of survival was performed using a log-rank (Mantel–Cox) test with a 95% confidence interval. Statistical analysis of antibody/neutralisation titres and viremia was performed using the Kruskal–Wallis test with Dunn’s correction for multiple comparisons. Significance levels: ^*^0.05, ^**^0.01, ^***^0.001, and ^****^< 0.0001.

## Data Availability

Raw data underpinning the figures associated with this paper are available in the Enlighten repository at http://dx.doi.org/10.5525/gla.researchdata.1943.
